# A Visual Distance-Based Capillary Immunoassay Using Biomimetic Polymer Nanoparticles for Highly Sensitive and Specific C-Reactive Protein Quantification

**DOI:** 10.3390/ijms25189771

**Published:** 2024-09-10

**Authors:** Ruodong Huang, Zhenbo Liu, Xinlin Jiang, Junqi Huang, Ping Zhou, Zongxia Mou, Dong Ma, Xin Cui

**Affiliations:** Key Laboratory of Biomaterials of Guangdong Higher Education Institutes, Department of Biomedical Engineering, Jinan University, Guangzhou 510632, China; cathyh1992@outlook.com (R.H.); calh8691@gmail.com (Z.L.);

**Keywords:** distance-based immunoassay, C-reactive protein, biomimetic nanoparticles, specificity

## Abstract

The low-cost daily monitoring of C-reactive protein (CRP) levels is crucial for screening acute inflammation or infections as well as managing chronic inflammatory diseases. In this study, we synthesized novel 2-Methacryloyloxy ethyl phosphorylcholine (MPC)-based biomimetic nanoparticles with a large surface area to develop a visual CRP-quantification assay using affordable glass capillaries. The PMPC nanoparticles, synthesized via reflux precipitation polymerization, demonstrated multivalent binding capabilities, enabling rapid and specific CRP capture. In the presence of CRP, PMPC nanoparticles formed sandwich structures with magnetic nanoparticles functionalized with CRP antibodies, thereby enhancing detection sensitivity and specificity. These sandwich complexes were magnetically accumulated into visible and quantifiable stacks within the glass capillaries, allowing for the rapid, sensitive, and specific quantification of CRP concentrations with a detection limit of 57.5 pg/mL and a range spanning from 0 to 5000 ng/mL. The proposed visual distance-based capillary biosensor shows great potential in routine clinical diagnosis as well as point-of-care testing (POCT) in resource-limited settings.

## 1. Introduction

C-reactive protein (CRP) is a pivotal biomarker in the acute phase response of the innate immune system, commonly associated with inflammation, infection, and tissue injury [1,2]. During episodes of infection or inflammation, CRP levels can increase dramatically, by up to 1000-fold. Initially, CRP binds to cell membrane components like phosphorylcholine and subsequently activates the complement system or exerts regulatory effects, thereby acting as a defense molecule against specific pathogens. CRP also exhibits activities similar to immunoglobulins (IgG), including the promotion of aggregation, phagocytosis, bacterial opsonization, and the precipitation of both cationic and anionic compounds. Extensive prospective studies have identified CRP as a critical biomarker for infections, inflammation, cardiovascular diseases, atherosclerosis, and related conditions [3,4]. Clinical guidelines from the American Heart Association and the Centers for Disease Control and Prevention (AHA/CDC) recommend biweekly CRP measurements [5], with levels below 1 μg/mL indicating low cardiovascular risk, 1 to 3 μg/mL indicating moderate risk, and levels exceeding 3 μg/mL indicating high risk. For example, CRP levels greater than 10 μg/mL are indicative of clinically evident inflammation, primarily caused by infection. Additionally, CRP holds potential as a therapeutic target for cardiac protection in acute myocardial infarction and neuroprotection in stroke cases [6]. Given its ability to reflect both the presence and severity of inflammation or infection, the cost-effective daily monitoring of CRP proves invaluable for the screening and management of these diseases.

Rapid point-of-care testing (POCT) methods have emerged for CRP application in clinical settings, particularly for managing bacterial infections at the bedside [7,8]. However, these methods often exhibit lower sensitivity, necessitating improvements in detection limits and ranges. Conventional CRP-quantification methods typically rely on immunoturbidimetry with a detection range of 3 to 8 μg/mL [9]. Distance-based measurements offer an alternative quantitative approach to traditional POCT devices, where visual signal-length variations are proportional to the target concentration [10]. Common methods include biochemical reactions that convert molecular signals into color bands, with the length of band corresponding to target molecule quantities [11,12]. Another approach involves measuring volume changes from gas generation reactions, which correlate with target molecule concentrations [13,14]. Recent innovations include detection methods that utilize changes in water permeability, where the target concentration correlated with liquid movement distances [15,16,17]. While these methods offer broad applications in detection targets through specific reagent and chip design modifications, challenges such as the complex operational procedures, reagent stability, and consistency across diverse detections hinder their clinical applications. Particularly for visual CRP detection [12,18,19,20], these methods generally achieve detection limits and ranges predominantly at the μg/mL level [21]. Furthermore, these technologies often necessitate high-resolution photolithographic microfabrication, thereby escalating costs and limiting widespread adoption in resource-constrained regions. In previous studies [21], we have developed a novel two-step competitive method for highly sensitive and broad-range CRP detection, with a limit of detection (LOD) of approximately 32 pg/mL and a detection range from 100 pg/mL to 10 µg/mL. However, challenges persist regarding cost and detection time due to the use of expensive reagents, such as antibodies, and multiple incubation steps.

Polymeric materials, known for their diversity and customizable production, offer significant cost advantages in various applications, including CRP detection [22,23]. CRP ligand materials can be classified into three major categories [24]: (1) compounds containing phosphorylcholine or related structures, (2) polycationic compounds such as poly-lysine and protamine sulfate, and (3) carbohydrates containing D-galactose-related structures. Studies have demonstrated that compounds like C-polysaccharide (CPS) and phosphoethanolamine can selectively bind CRP from body fluids in the presence of calcium ions. However, significant non-target proteins, such as human serum albumin (HSA) and immunoglobulin M (IgM), are often present in the eluates [25]. Recently, 2-(methacryloyloxy)ethyl phosphorylcholine (MPC) polymers, which mimic biomembrane structures, have garnered increasing attention [26]. The U.S. Food and Drug Administration has approved their use as surface coatings for various medical devices, including vascular stents, guidewires, and contact lenses [27]. Previous research indicates that, compared to other phosphorylcholine derivatives, 2-methacryloyloxyethyl phosphorylcholine exhibits higher specificity for CRP binding [28]. Chanika et al. described a paper-based electrochemical sensor that utilizes thiol-terminated poly(2-methacryloyloxyethyl phosphorylcholine) (PMPC-SH) [29]. This polymer self-assembles onto gold nanoparticles on screen-printed electrodes via thiol groups, effectively detecting CRP in human serum without interference from bilirubin, myoglobin, and albumin. Matsuura et al. developed a plasmonic chip with an MPC polymer ligand layer for CRP detection, achieving a detection limit of approximately 230 pg/mL [30]. Kitayama et al. employed MPC-coated gold nanoparticles as the CRP recognition layer, using localized surface plasmon resonance to achieve a detection limit of about 50 ng/mL [31]. María et al. reported a grating-based biosensor using MPC-responsive hydrogels and a simple laser-based optical setup for label-free CRP sensing, with a detection limit of 1.07 mg/L in diluted human serum [32]. Despite the exceptional biocompatibility and hydrophilicity of MPC polymers [33,34,35], which reduce non-specific adsorption, challenges remain in improving specificity and sensitivity in biosensor applications. Moreover, these technologies often require micro-/nano-fabrication facilities and specialized optical measurement platforms, which can limit their detection performance and anti-interference capabilities.

Nanoparticles or nanogels (NPs), which are submicron-sized, three-dimensional crosslinked polymer networks, have shown increasing promise in various biosensors due to their controllable size, high surface area, stable internal structure, and customized responsive properties [36,37]. Vishnu et al. developed semi-interpenetrating NPs of chitosan (CS) and poly(methacrylic acid) (PMAA). The deprotonated CS-PMAA, combined with gold NPs, acts as a catalyst and exhibits excellent detection performance for the anti-leukemia drug 5-fluorouracil on modified electrodes, achieving a detection limit of 1.6 ng/mL [38]. Daniel et al. developed reactive and fluorescent glucose oxidase NPs with surface phosphate groups coordinated with Ce(III) cations, achieving a detection limit of 73.37 nM [39]. Recently, an NP-based surface plasmon resonance (SPR) platform was developed via radical precipitation polymerization, enabling clinical SARS-CoV-2 NAb detection without complex pretreatment [40]. Due to the multivalent binding capability between NPs and target proteins, this method showed significantly enhanced SPR signals. However, the reliance on large and costly signal measurement devices such as SPR instruments and fluorometers, along with cumbersome washing and incubation steps, restricts their further development as a POCT technique.

To our knowledge, the use of PMPC NPs as CRP bioreceptors for POCT applications, without the need for large equipment, has not yet been demonstrated, despite their potential to significantly reduce detection costs and improve robustness and stability [41]. In this work, we coupled the magnetic nanoparticles with CRP antibodies, so that the nanoparticles have the function of the specific recognition and binding of the CRP protein. After the phosphatidylcholines polymer specifically binds CRP, the magnetic nanoparticles and the polymer form a larger complex through the combination of an antigen and an antibody. Finally, the aggregation is quantified into a visual strip under the magnetic field. We employed PMPC NPs and antibody-functionalized magnetic beads to achieve simple, visual and specific distance-based CRP quantification. This method substantially reduces detection costs compared to traditional antibody-based immunoassays and enables the rapid, naked eye-based quantification of CRP levels without requiring large detection equipment.

## 2. Results

### 2.1. Principle of PMPC NPs-Based Immunoassay for Visual CRP Quantification

**Figure 1:**
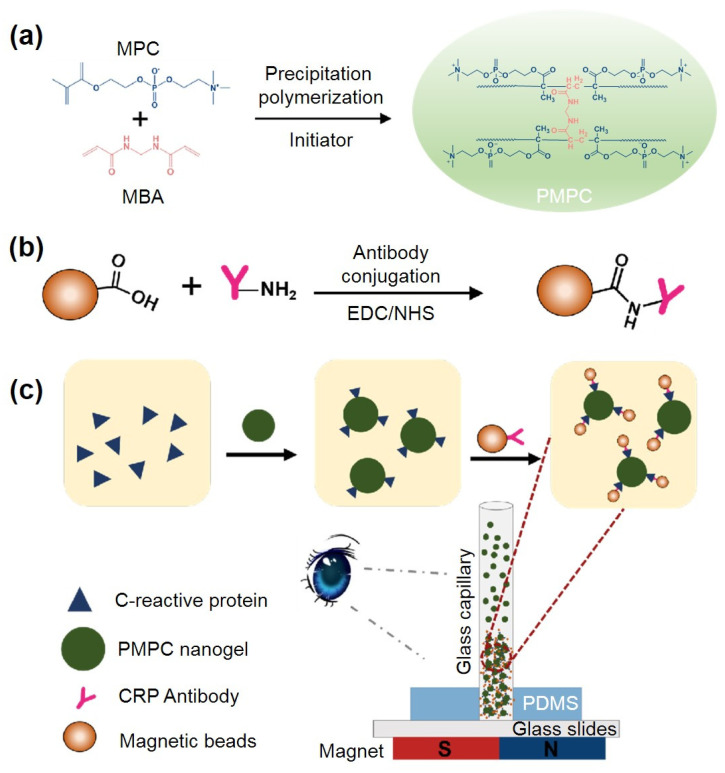
(**a**) Illustration of the preparation of PMPC nanoparticles (NPs) using 2-Methacryloyloxyethyl phosphorylcholine (MPC)and N,N-methylene-bis-acrylamide (MBA). (**b**) Illustration of the preparation of antibody-conjugated magnetic NPs using EDC/NHS. (**c**) Schematic diagram of the developed PMPC NPs-based immunoassay for visual distance-based C-reactive protein (CRP) quantification with visible and quantifiable stacks within the glass capillaries.

A derivative of phosphorylcholine, 2-Methacryloyloxyethyl phosphorylcholine (MPC), has garnered significant attention due to its exceptional biocompatibility, which stems from its zwitterionic functional groups at both ends. It is increasingly recognized as a high-affinity artificial ligand for C-reactive protein (CRP) in the presence of calcium ions [28]. Inspired by these properties, we have developed a visual quantitative CRP detection approach based on biomimetic PMPC nanoparticles (NPs) and low-cost glass capillaries (Figure 1). A novel cell-membrane biomimetic PMPC microsphere was synthesized using precipitation polymerization with a highly hydrophilic crosslinker: N,N′-methylenebisacrylamide (MBA). This design enhances CRP enrichment due to the significantly higher surface-area-to-volume ratio and faster diffusion and interaction with target molecules compared to bulk materials. Subsequently, the PMPC NPs were interconnected using CRP antibody-functionalized magnetic beads, enabling the visualization of various CRP concentrations. Moreover, the incorporation of functionalized magnetic beads significantly improved detection specificity. Upon the presentation of CRP antigens, a sandwich structure forms between the magnetic particles, PMPC NPs, and CRP. Assisted by a magnetic field, this complex aggregates within the low-cost glass capillary, producing a visually quantifiable bar whose length correlates with CRP concentration. Owing to the porous three-dimensional structure of PMPC NPs, their large surface area, and abundant recognition sites facilitating multivalent binding, this method is anticipated to achieve rapid, highly sensitive and specific CRP quantification.

### 2.2. Synthesis and Characterization of the PMPC NPs and Antibody-Functionalized NPs

**Figure 2:**
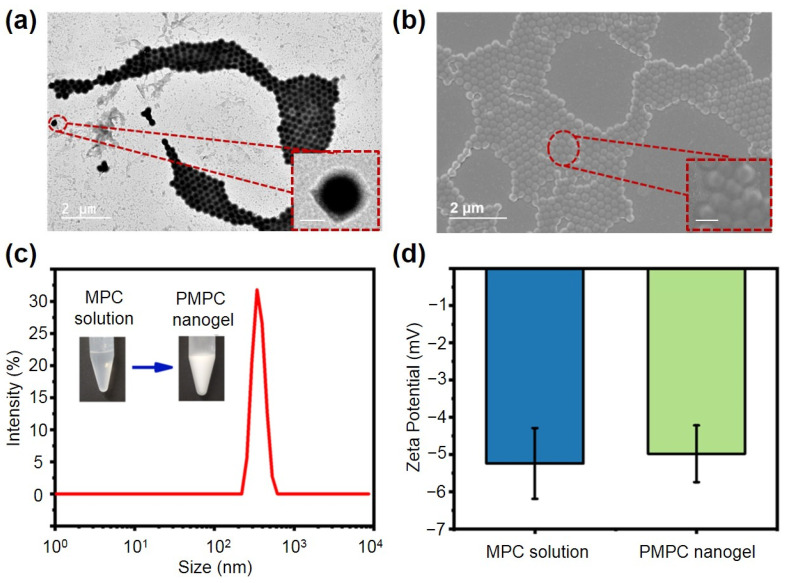
(**a**) TEM images of PMPC NPs. Scale bar for insert image is 100 nm. (**b**) SEM image of PMPC NPs. Scale bar for insert image is 200 nm. (**c**) Hydrodynamic sizes of PMPC NPs. (**d**) Zeta potentials of MPC and PMPC NPs.

To enhance surface hydrophilicity and reduce non-specific protein adsorption, we synthesized PMPC NPs using a reflux precipitation polymerization technique. This method is notable method for synthesizing uniform microspheres without the need for surfactants or stabilizers [42,43]. When MPC dissolved in acetonitrile undergoes polymerization initiated by AIBN at high temperatures, this leads to the formation of nanogels that precipitate out of the solution due to their insolubility in acetonitrile (Figure 1a). We examined the morphology and structure of the synthesized NPs using transmission electron microscopy (TEM). As shown in Figure 2a,b, the prepared NPs were spherical and uniform with an average diameter of approximately 160 nm. Scanning electron microscopy (SEM) results further confirmed the sphere shape and uniform size of the synthesized PMPC NPs. We also characterized the size and zeta potential of PMPC NPs using dynamic light scattering (DLS). As shown in Figure 2c, MPC, as a zwitterion, is highly soluble in water, resulting in a clear, colorless solution. After precipitation polymerization, the resulting NPs are insoluble in water, appearing as a milky-white suspension. Notably, the PMPC NPs exhibited good dispersibility and stability in pure water without sedimentation over time. The DLS results show that the NPs had a uniform size distribution with an average hydrodynamic diameter of approximately 503 nm. Moreover, upon polymerization, the zeta potential of MPC changed slightly from −5.24 mV to −4.98 mV (Figure 2d), indicating the phosphate groups on the PMPC surface were unaffected by the polymerization process.

**Figure 3:**
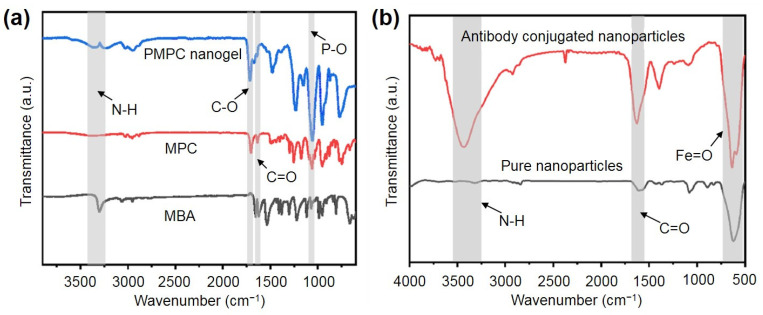
(**a**) FTIR spectra of PMPC NPs, MPC and MBA. (**b**) FTIR spectra of antibody-modified magnetic beads and pure nanoparticles.

To further characterize the successful synthesis of PMPC NPs, FTIR spectra (Figure 3a) of PMPC NPs, MPC, and MBA are examined. The crosslinker MBA shows an absorption peak at 3302 cm⁻^1^ corresponding to the secondary amide bond (-NH), which diminishes after crosslinking with MPC. The C-O characteristic absorption peaks from the MPC ester bond and the P-O absorption peaks from the phosphate group are observed at 1703 cm⁻^1^ and 1100 cm⁻^1^, respectively. Additionally, the PMPC NPs exhibit the C=O absorption peak from both MBA and MPC at 1623 cm⁻^1^. Hence, these results demonstrated the successful synthesis of the PMPC NPs.

To enhance the specificity for CRP antigen recognition, we further prepared antibody-functionalized magnetic beads via an amide reaction (Figure 1b). As shown in Figure 3b, the antibody-functionalized beads exhibit clear peaks of amide bonds at 3488 cm⁻^1^ and 1546 cm⁻^1^, corresponding to -NH and -C=O, respectively. The absorption peak at 545 cm⁻^1^ of -Fe=O, from ferric oxide, can be also observed in the functionalized NPs, confirming that the antibodies were successfully conjugated to the magnetic beads.

### 2.3. Performance of the PMPC NPs for Binding CRP

**Figure 4:**
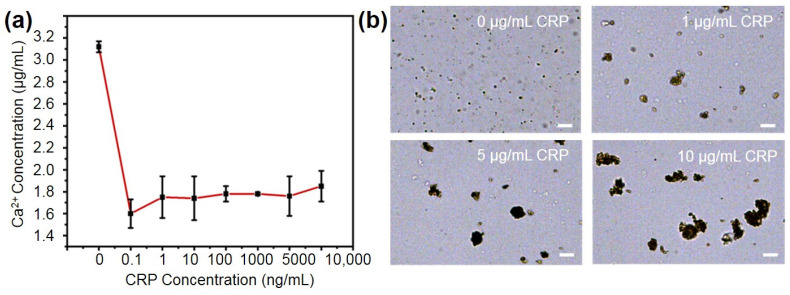
(**a**) Residual calcium-ion content in supernatant after incubation PMPC NPs with CRP. (**b**) Representative microscope images of PMPC–CRP–magnetic beads complexes after incubation with different concentrations (0 μg/mL, 1 μg/mL, 5 μg/mL, 10 μg/mL) of CRP. The scale bar is 20 μm.

To evaluate the performance of the synthesized PMPC NPs in recognizing CRP, we incubated PMPC NPs with various concentrations (0 μg/mL, 1 μg/mL, 5 μg/mL, 10 μg/mL) of CRP and then evaluated the residual CRP and calcium ions in the supernatant. Since CRP is composed of five globular subunits that can each bind two calcium ions [44], the binding of CRP to PMPC NPs would be expected to consume calcium ions. As shown in Figure 4a, the calcium-ion content in the supernatant decreased from 3.12 μg/mL to ~1.7 μg/mL following the addition of CRP, compared to the blank control. However, there was no significant difference in calcium ion content across the different CRP concentrations. This lack of variation is likely due to the excess of calcium ions relative to CRP, with a molar ratio difference of 10,000 times, leading to the saturation of CRP–calcium ion chelation.

To further assess the feasibility of using PMPC NPs for the visual detection of CRP antigens, we incubated different concentrations (0, 1 μg/mL, 5 μg/mL, 10 μg/mL) of CRP solutions with nanoparticles and magnetic beads at room temperature for 1 h. As shown in Figure 4b, upon binding with CRP, the PMPC NPs aggregated to form irregularly shaped complexes, with the particle size increasing proportionally to the CRP concentration. The diameter of the complexes increased from 500 nm to >20 μm, enabling the subsequent visual quantification of CRP concentrations. This phenomenon is possibly attributed to the presence of multiple antibodies and recognition sites on the surface of both the magnetic beads and the spherical PMPC NPs, which promotes the complex formation with the presence of CRP.

### 2.4. Optimization of Experimental Conditions

**Figure 5:**
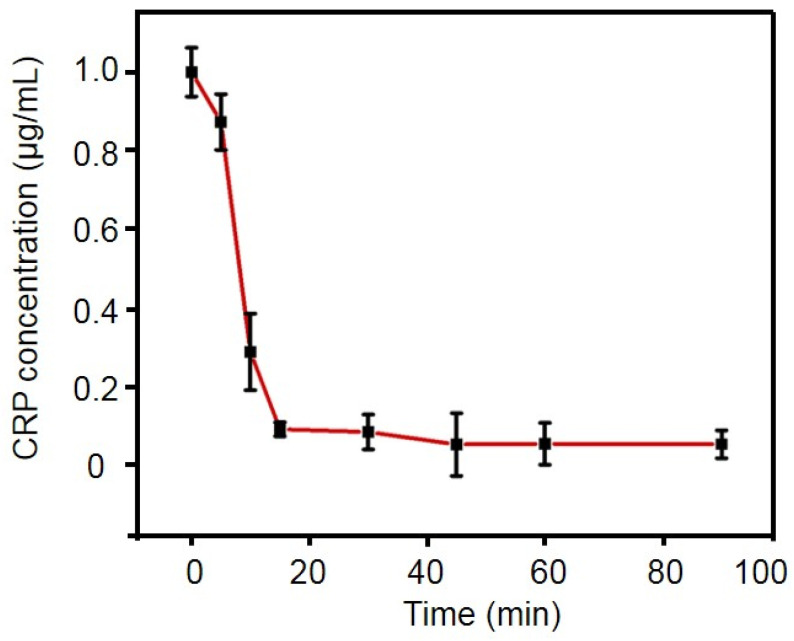
The capture efficiency of CRP using PMPC NPs under different incubation times.

To achieve a high sensitivity in CRP quantification using the PMPC NPs, we optimized the incubation time to capture CRP by employing a commercially available CRP ELISA kit at various incubation intervals. The initial CRP concentration in all test solutions was 1 μg/mL. As shown in Figure 5, after 5 min of incubation, the PMPC nanogel captured approximately 12.64% of the CRP in the solution. The capture efficiency significantly increased to 71.08% after 10 minutes’ incubation, representing the highest rate of increase, likely due to the rapid recognition and capture capabilities of the nanogel. As the incubation time extended, the capture efficiency exceeded 90% after 30 min, with a slight increase from 91.4% to 94.6% between 30 and 45 min. After 45 min, the capture efficiency plateaued at ~95%, suggesting that the binding of CRP on the PMPC nanogel had reached saturation. Therefore, to achieve optimal detection performance with PMPC nanogel in subsequent assays, a reaction time of 45 min was chosen, which was much faster compared to traditional immunoassays that typically require several hours [21]. Moreover, it is noteworthy that in scenarios requiring rapid CRP quantification, the incubation time can be shortened to 15 min, during which approximately 90% of CRP can be captured. Although the capture efficiency is lower than that after 30 min, it still provides a relatively rapid and sensitive detection approach for CRP quantification.

### 2.5. Linearity Range and Limit of Detection

**Figure 6:**
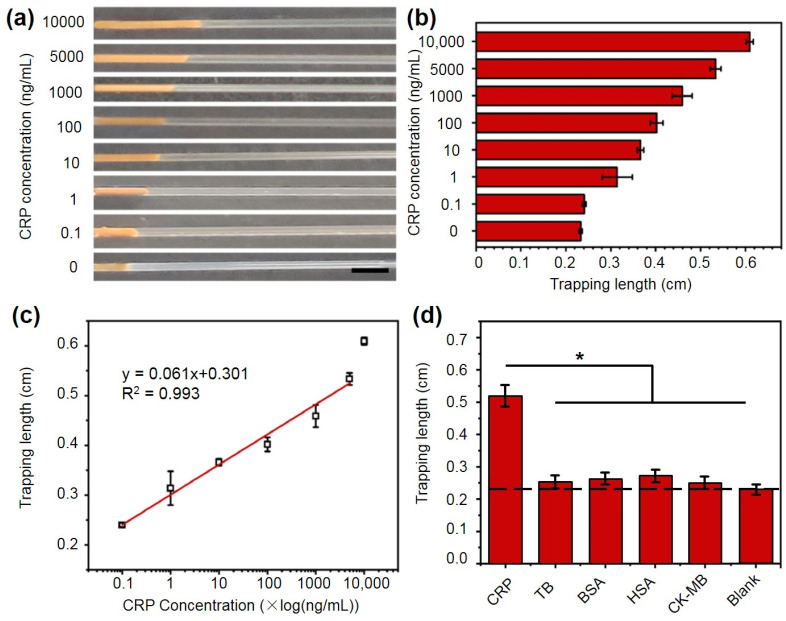
(**a**) The representative images of the visually quantifiable strip in glass capillary tubes. The scale bar is 2 mm. (**b**) The accumulation distance of different concentrations of CRP in the glass chip was quantified using ImageJ software (v1.50i). (**c**) Correlation of accumulation distance and CRP concentration. (**d**) The quantified accumulation length in glass capillary chips with different interfering substances such as BSA, HSA, TB and CK-MB (5 µg/mL). Each data point is calculated from at least three replicate measurements, and the error bars indicate the standard deviations. * *p* < 0.05.

In the presence of the CRP antigen, a sandwiched structure formed due to the specific antigen/antibody interaction. The target CRP binds at one end to the biomimetic PMPC nanoparticles (NPs) and at the other end to magnetic beads modified with anti-CRP antibodies. Under magnetic force, the complex rapidly accumulates into a visually quantifiable strip within a glass capillary tube. The distance of the strip was measured using ImageJ (v1.50i) software, which is expected to vary with different CRP concentrations. We then explored the relationship between CRP concentrations and the accumulation distance. As shown in Figure 6a–c, the higher the CRP concentration, the longer the accumulation strip. When the CRP concentration increased from 100 pg/mL to 10 μg/mL, the strip length increased from 0.23 mm to 0.61 mm. A linear positive correlation was observed in the CRP concentration range of 0–5000 ng/mL with a regression equation of y = 0.061x + 0.301 and R^2^ = 0.993. To determine the limit of detection (LOD), the standard deviation of the lowest concentration within the lower detection range (0–10 ng/mL) was multiplied by three and divided by the slope of the fitted equation (Figure S1). The LOD was found to be ~57.5 pg/mL. Therefore, this method exhibits high sensitivity and a broad detection range for CRP quantification as compared to the previous studies (Table S2) [45,46,47,48], likely due to the large specific surface area and abundant multivalent recognition sites of PMPC nanoparticles, which enhances the collision probability between CRP and NPs and leads to efficient binding [49]. Further evaluation (Table S1) using a commercial ELSIA kit confirmed the precise and repeatable performance of the proposed assay. Additionally, it is noteworthy that the proposed glass capillary device (Table S2) has a cost of approximately USD 0.01, making it more economically feasible than other point-of-care testing (POCT) devices based on enzymes, gas expansion, hydrogel permeability, and commercially available materials [12,18,19,20]. Overall, the proposed biomimetic PMPC nanoparticle-based visual CRP-quantification method offers higher binding capacity, a shorter processing time, and lower cost.

### 2.6. Specificity Experiments

Signal specificity is a crucial factor for evaluating sensor performance. To validate the specificity of the assay, we measured accumulation distance in glass capillary chips in the presence of potential interfering substances, including bovine serum albumin (BSA), human serum albumin (HSA), thrombin (TB) and creatine kinase-MB (CK-MB). BSA was chosen due to its structural similarity to HSA, which is the most abundant protein in human blood plasma. TB, on the other hand, is a key enzyme involved in inflammatory responses. Additionally, CK-MB is a crucial biomarker for the diagnosis and monitoring of heart failure and myocardial infarction. The results (Figure 6d and Figure S2) showed that the capillary accumulation lengths for these interfering substances (5 µg/mL) were nearly identical to the length (~0.23 mm) observed with 0 µg/mL CRP (blank). In contrast, the accumulation length observed with 5 µg/mL CRP was significantly longer (~0.54 mm)—approximately 2.3 times greater than that observed with the interfering substances. These findings indicate that the visual distance-based quantification method for CRP based on PMPC nanoparticles exhibits excellent selectivity and specificity, making it suitable for stable operation in complex environments.

## 3. Discussion

Rapid point-of-care testing (POCT) methods for CRP have emerged as essential tools in clinical settings, particularly for managing bacterial infections at the bedside. However, these methods often suffer from lower sensitivity, necessitating improvements in detection limits and ranges. Conventional CRP-quantification methods, such as immunoturbidimetry, typically exhibit a detection range spanning from 3 to 8 μg/mL [12,18,19,20]. This limitation reduces their effectiveness in detecting lower concentrations of CRP, which are critical for the early diagnosis and monitoring of inflammatory conditions. Distance-based quantification methods present an alternative to traditional POCT devices. Despite their innovative nature, these methods often achieve detection limits and ranges predominantly at the μg/mL level, which is insufficient for highly sensitive CRP detection [13,14]. Furthermore, the complex operational procedures, reagent stability issues, and inconsistencies across diverse detection scenarios hinder their clinical applications.

In our study, we developed a sensitive and specific CRP-quantification assay using PMPC nanoparticles functionalized with CRP recognition moieties and antibody-functionalized magnetic particles. This method demonstrates several key advantages over previous work. Firstly, our assay achieved an enhanced sensitivity, with a limit of detection of 57.5 pg/mL and a broad detection range of 0–5000 ng/mL. This improvement is contributed to both PMPC nanoparticles’ high specificity for CRP binding and non-specific adsorption [25,27], as well as the use of antibody-conjugated magnetic NPs. In addition, by employing readily available glass capillaries and PMPC nanoparticles, our assay reduces manufacturing complexity and costs [15,16,17]. This makes it a viable alternative for widespread clinical applications, particularly in resource-limited settings where cost-effective solutions are crucial. Furthermore, the visual, distance-based quantification approach eliminates the need for sophisticated equipment and complex operational procedures [11,12]. This further enhances the assay’s accessibility and ease of use, enabling rapid and efficient CRP detection at the point of care. Hence, the proposed assay partially addresses some limitations of existing CRP-quantification techniques by combining the advantages of PMPC nanoparticles with a simple, cost-effective, and highly sensitive detection platform. This approach holds significant potential for enhancing CRP detection capabilities in clinical and field settings, paving the way for broader adoption and improved patient outcomes.

While our current study demonstrates promising results, several avenues for future research could further enhance the assay’s capabilities. As serum CRP is a sensitive and stable marker of inflammation [12,18,19,20], incorporating pre-processing steps for whole-blood samples could improve detection accuracy by minimizing matrix effects, thereby enhancing the assay’s robustness in real-world applications [21,30]. Developing a portable centrifuge system would facilitate point-of-care testing by allowing rapid and efficient sample processing directly at the bedside, further reducing the assay’s turnaround time. Additionally, extending the assay to include the multiplex detection of CRP and other biomarkers could provide comprehensive diagnostic information, beneficial for managing complex diseases where multiple biomarkers are involved [29]. Conducting field tests in resource-limited settings would provide valuable insights into the assay’s practicality and reliability in diverse environmental conditions, paving the way for global implementation [27,30,32]. Incorporating dynamic testing conditions that mimic physiological environments, such as simulating blood flow, can better validate the sensor’s performance in real-world scenarios. Additionally, evaluating the long-term stability of the biosensor under various conditions (temperature, pressure, pH and biotic factors such as enzymes) will be crucial to ensure its viability for routine clinical and point-of-care applications for serum samples, although our preliminary experiments demonstrate a good material stability within one month (Figure S3). These enhancements will refine the sensor’s design and further validate its reliability and effectiveness.

In conclusion, our study presents a novel, efficient, and cost-effective approach to CRP quantification, addressing the limitations of existing methods while offering a practical solution for real-world applications (Table S2). The PMPC nanoparticles, synthesized via reflux precipitation polymerization, exhibited multivalent binding capabilities, which facilitated the rapid and precise capture of CRP. By forming sandwich complexes with CRP antibody-conjugated magnetic nanoparticles, we achieved a detection limit of 57.5 pg/mL and a broad detection range up to 5000 ng/mL. We envision that the developed PMPC NPs-based visual CRP-quantification assay is a potential low-cost alternative to traditional CRP detection methods for complex biological samples.

## 4. Materials and Methods

### 4.1. Materials

Ferric chloride hexahydrate (FeCl_3_·6H_2_O), sodium acetate (NaAc), 1-Ethyl-3-(3-dimethylaminopropyl) carbodiimide (EDC), N-hydroxysuccinimide (NHS), N,N′-Methylenebisacrylamide (MBA), 2,2-azobisisobutyronitrile (AIBN, 99%), acetonitrile and tri-sodium citrate were provided by Aladdin Industrial Co., Ltd. (Shanghai, China). Furthermore, 2-Methacryloyloxyethyl phosphorylcholine (MPC) was purchased from Sigma-Aldrich (St. Louis, MO, USA). The monoclonal antibody to C-reactive protein (anti-CRP, Homo sapiens, catalog number: MAA821Hu22), native C-reactive protein (CRP, Homo sapiens, catalog number: NPA821Hu02) and an ELISA Kit for Creatine Kinase MB Isoenzyme (CKMB, catalog number: SEA479Mu) were purchased from Cloud-Clone Co., Ltd. (Wuhan, China). Phosphate-buffered saline (PBS, 1×) was purchased from Cytiva (Marlborough, MA, USA), and bovine albumin (BSA) were purchased from Jetway Co., Ltd. (Guangzhou, China). A Human C-Reactive Protein ELISA Kit (RK00078) was purchased from Abclonal Co., Ltd. (Wuhan, China). A Calcium Colorimetric Assay Kit was obtained from Beyotime Biotechnology (Shanghai, China). All chemical compounds were used as received without further purification unless otherwise stated.

### 4.2. Instrumentation

A scanning electron microscope (JEOL JSM-6700F; JEOL, Ltd., Tokyo, Japan) and a transmission electron microscopy (JEOL JEM-2010; JEOL, Ltd., Tokyo, Japan) were used to study the morphology of synthesized PMPC nanoparticles. The zeta potential and hydrodynamic particle size of different samples were measured using a Zetasizer Nano ZS (Mallern Instruments, Worcestershire, UK). Fourier transform infrared spectroscopy (FTIR) was used to examine the NPs using a Nexus 670 FT-IR spectrophotometer (Nicolet Instrument Corporation, Madison, WI, USA) in transmittance mode with KBr plates. A multimode microplate reader (BioTek Synergy H1; BioTek Instruments Inc., Winooski, VT, USA) was used for ELISA experiments.

### 4.3. Synthesis and Characterization of PMPC Nanoparticles

A total of 340 mg of MPC, 30 mg of MBA, and 56 mg of AIBN was accurately weighed and dissolved in 40 mL of acetonitrile via sonication for 20 min. The solution was subsequently filtered through a 0.2 μm filter and transferred to a 150 mL reaction flask. The reaction flask with a reflux condenser was immersed in an oil bath maintained at 90 °C under a nitrogen atmosphere for 1 h. Then the mixture was allowed to cool to room temperature. Unreacted monomers and crosslinking agents were removed by centrifugation at 11,000 RPM for 15 min. The precipitate was washed three times with deionized water to further eliminate residual acetonitrile. The final precipitate was then dispersed in 3 mL of deionized water and stored at 4 °C for subsequent use. Note that the reaction should be performed in a fume hood with appropriate personal protective equipment to ensure safety.

PMPC nanoparticles, MBA and MPC were vacuum-dried, and then the sample was mixed with KBr, separately, followed by pressing in a dense plate. Infrared spectra were obtained using a FT-IR spectrophotometer in transmittance mode with KBr plates. Each sample was examined in at least three independent scans in a range from 4000 to 500 cm^−1^ at a resolution of 4 cm^−1^.

Surface morphologies were analyzed using a scanning electron microscope (SEM). The prepared nanoparticle solution was dripped onto a glass slide and allowed to dry naturally. After drying, the sample was coated with a thin layer of gold via sputtering. The gold-coated sample was then observed using SEM to capture images of the surface morphology.

Transmission electron microscopy (TEM) was used to further characterize the surface morphology of the nanoparticles. The nanoparticle solution was dripped onto a 200-mesh copper grid and allowed to dry naturally. Once dried, the sample was observed using TEM for detailed imaging of the nanoparticle surfaces.

### 4.4. Synthesis and Characterization of Antibody-Conjugated Magnetic Nanoparticles

Magnetic Fe_3_O_4_ nanoparticles functionalized with carboxyl groups were synthesized following a previous report [21]. Briefly, 1.2 g of anhydrous sodium acetate, 0.2 g of trisodium citrate, and 0.65 g of ferric chloride hexahydrate were dissolved in 20 mL of ethylene glycol under vigorous stirring for 30 min. The resulting mixture was then transferred to a stainless-steel autoclave and subjected to hydrothermal treatment at 200 °C for 10 h. After cooling to room temperature, the product was washed sequentially with different ethanol solutions (25%, 50%, 75%, and 100%) to remove impurities. After hydrothermal treatment, Fe_3_O_4_ has magnetic properties, so the product and supernatant can be separated under the action of a magnetic field. No additional centrifugation is required. Finally, the nanoparticles were dried in a vacuum oven at 50 °C and stored in a dark, dry environment for further use.

Fe_3_O_4_ nanoparticles (0.5 mg) were ultrasonically dispersed in 1 mL of MES buffer (pH 6.0). Subsequently, 100 mM EDC and 100 mM NHS were added, and the mixture was shaken for 30 min to activate the carboxyl groups. After activation, the nanoparticles were washed three times with PBS buffer (pH 7.4) to remove unreacted EDC and NHS. The activated nanoparticles were then resuspended in 1 mL of PBST, and 80 μL of anti-CRP antibody solution was added. The reaction was carried out at room temperature for 4 h in the dark. Then, the nanoparticles were washed three times with PBS buffer (pH 7.4), blocked with 1% BSA solution overnight at 4 °C and used within one month based on the previous report [21]. Finally, the nanoparticles were resuspended in 200 μL of PBS buffer. The FT-IR experiments of magnetic nanoparticles and antibody-conjugated magnetic nanoparticles were similar with that for PMPC NPs.

### 4.5. Optimization of Incubation Time

Then, 95 μL of Tris-CaCl_2_ solution (10 mM Tris, 140 mM NaCl, 2 mM CaCl_2_, pH 8.0) and 5 μL of PMPC nanoparticles were added to each tube. After mixing, 200 μL of different concentrations (0 μg/mL, 1 μg/mL, 5 μg/mL, 10 μg/mL) of CRP solution was added. The reaction was allowed to proceed for various intervals (0, 5 min, 10 min, 15 min, 30 min, 45 min, 60 min, 90 min). At each time, the mixture was centrifuged at 5000 RPM for 15 min. The supernatant was collected for the subsequent analysis of calcium-ion concentration and CRP concentration using the specific kits.

### 4.6. Microscope Imaging of Nanoparticle Complex

Subsequently, 200 μL of different concentrations (0 μg/mL, 1 μg/mL, 5 μg/mL, 10 μg/mL) of CRP solution, 5 μL of PMPC nanoparticles solution, and 95 μL of Tris-CaCl_2_ were mixed and incubated for 45 min. After incubation, the mixtures were centrifuged at 5000 RPM for 15 min to remove the supernatant. The precipitate was washed with pure water, followed by adding 10 μL of antibody-conjugated magnetic NPs, and incubated for 1 h. After incubation, the mixture was imaged using a microscope.

### 4.7. Detection of CRP Concentrations Using PMPC NPs

To explore the sensitivity of the assay, different concentrations (10,000 ng/mL, 5000 ng/mL, 1000 ng/mL, 100 ng/mL, 10 ng/mL, 1 ng/mL, 0.1 ng/mL, 0 ng/mL) of CRP solutions (200 μL), PMPC NPs solution (5 μL), and Tris-CaCl_2_ (95 μL) were mixed and incubated for 45 min. The mixtures were then centrifuged at 5000 RPM for 15 min and washed three times with pure water to remove unbound CRP. Subsequently, 10 μL of antibody-conjugated magnetic NPs was added and incubated for 1 h. The resulting PMPC–CRP–magnetic particle complexes were then injected into a glass capillary tube, which was inserted into a hole of PDMS bonded to a microscope slide. Then, a magnet was used to accumulate the PMPC–CRP–magnetic particle complexes in the capillary tube, and a visual strip could be observed and quantified using ImageJ software (v1.50i).

Note that all materials are suggested to be stored at 4 °C, shaken well before use, and used within one month.

### 4.8. Specificity Experiment

To verify the specificity of the assay, three different interfering substances, including bovine serum albumin (BSA), human serum albumin (HSA), thrombin (TB) and creatine kinase-MB (CK-MB), were selected and tested under the optimal conditions for CRP detection. The concentration of the interference solution was 5 μg/mL. Other experimental conditions were the same as those used in the detection of CRP.

### 4.9. Statisticsusing

Three independent experiments were repeated for each study. Statistical significance (*, *p* < 0.05; **, *p* < 0.01) for multiple comparisons was examined by one-way ANOVA. Data were presented as mean ± SD.

## Data Availability

Data will be available when requested.

## Supplementary Materials

The following supporting information can be downloaded at: https://www.mdpi.com/article/10.3390/ijms25189771/s1.
