# Supplementary material for: A Visual Distance-Based Capillary Immunoassay Using Biomimetic Polymer Nanoparticles for Highly Sensitive and Specific C-Reactive Protein Quantification
Source: Int J Mol Sci. 2024 Sep 10;25(18):9771. doi: 10.3390/ijms25189771 (PMC11431823; doi:10.3390/ijms25189771)
Supplement: Supplementary file 1 [file ijms-25-09771-s001.zip › ijms-3159897-supplementary.pdf]

Supporting Information

A Visual Distance-Based Capillary Immunoassay Using Biomimetic Polymer Nanoparticles for Highly Sensitive and Specific C-Reactive Protein Quantification

Ruodong Huang †, Zhenbo Liu †, Xinlin Jiang, Junqi Huang, Ping Zhou, Zongxia Mou, Dong Ma * and Xin Cui *

Key Laboratory of Biomaterials of Guangdong Higher Education Institutes, Department of Biomedical Engineering, Jinan University, Guangzhou 510632, China; cathyh1992@outlook.com (R.H.); calh8691@gmail.com (Z.L.)

* Correspondence: tmadong@jnu.edu.cn (D.M.); cx2019@jnu.edu.cn (X.C.)

† These authors contributed equally to this work.

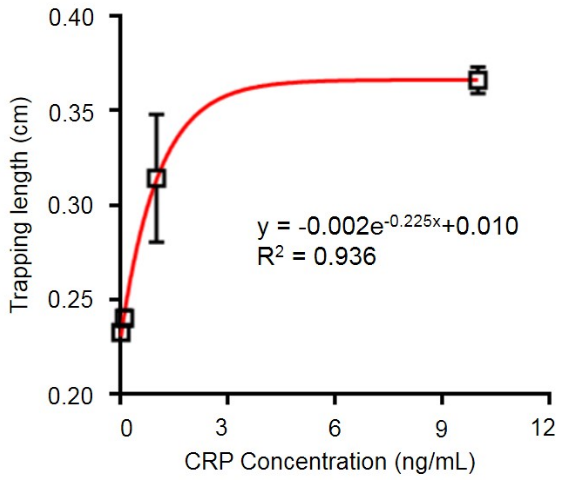

Figure S1. The correlation between the lower CRP concentrations (0-10 ng/mL) and the length of visually quantifiable strip in glass capillary tubes.

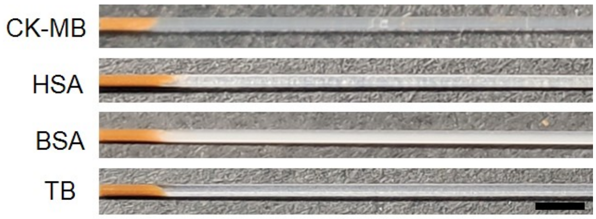

Figure S2. The representative images of the visually quantifiable strip in glass capillary tubes for specificity experiments. The scale bar is 2 mm.

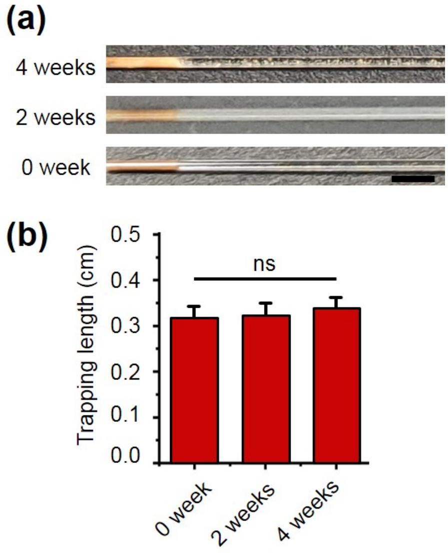

Figure S3. (a) The images of the visually quantifiable strip in glass capillary tubes using the synthesized PMPC and magnetic NPs after different storage periods for detecting CRP (1 ng/mL). The scale bar is 2 mm. (b) Quantified distance of visual strips in the glass chips.

Table S1. The comparation of measured results using both the developed assay and a commercial ELISA kit.

Sample	The standard concentration	Measured concentration using this assay	Measured concentration using ELISA
#1	10 ng/mL	9.4 ± 0.7 ng/mL (RSD=7.4%)	10.5 ± 0.6 ng/mL (RSD=5.7%)
#2	100 ng/mL	101 ± 3.1 ng/mL (RSD=3.1%)	103 ± 2.8 ng/mL (RSD=2.7%)
#3	10 μg/mL	10.2 ± 0.4 μg/mL (RSD=3.9%)	9.6 ± 0.2 μg/mL (RSD=2.1%)

Table S2. Comparison of the proposed CRP detection assay with previous reports.

Methods	Limit of detection	Detection range	Quantification equipment	Visual quantification	Ref.
Dual electrochemical magnetoimmunosensor	8 ng/mL	10 – 5,000 ng/mL	Electrochemical workstation	×	[1]
Paper-based electrochemical sensor	1.6 ng/mL	5 – 5000 ng/mL	Electrochemical workstation	×	[2]
Fluorescence polarization immunoassay	207 ng/mL	20000 – 100,000 ng/mL	Fluorescent imaging	×	[3]
Bio/chemical hybrid sensing system	10 pM	10-1000 pM	PMPC-grafted-plasmonic chips	×	[4]
Hydrogel-based label-free biosensor	0.3 mg/L	0.3-50 mg/L	Diffraction measurements	×	[5]
Immunochromatographic strips and gas	0.041 ng/mL	0.05 - 6.25 ng/mL	Microfabricated glass	√	[6]

generation biosensors			microfluidic chips		
Lateral flow immunoassays and AuNPs	100 ng/mL	100 – 5,000 ng/mL	Color or fluorescent imaging	√	[7]
PMPC-based distance assay	57.5 pg/mL	0.1 – 5,000 ng/mL	Affordable glass capillaries	√	This work

References

[1] A. g. Molinero-Fernández, M. Moreno-Guzman, L. Arruza, M. A. n. López, and A. Escarpa, "Toward early diagnosis of late-onset sepsis in preterm neonates: dual magnetoimmunosensor for simultaneous procalcitonin and C-reactive protein determination in diagnosed clinical samples," *ACS sensors,* vol. 4, pp. 2117-2123, 2019.

[2] C. Pinyorospathum, S. Chaiyo, P. Sae-Ung, V. P. Hoven, P. Damsongsang, W. Siangproh, *et al.,* "Disposable paper-based electrochemical sensor using thiol-terminated poly (2-methacryloyloxyethyl phosphorylcholine) for the label-free detection of C-reactive protein," *Microchimica Acta,* vol. 186, pp. 1-10, 2019.

[3] M. Fukuyama, M. Maeki, A. Ishida, H. Tani, A. Hibara, and M. Tokeshi, "One-step non-competitive

fluorescence polarization immunoassay based on a Fab fragment for C-reactive protein quantification," *Sensors and Actuators B: Chemical,* vol. 326, p. 128982, 2021.

[4] R. Matsuura, K. Tawa, Y. Kitayama, and T. Takeuchi, "A plasmonic chip-based bio/chemical hybrid sensing system for the highly sensitive detection of C-reactive protein," *Chemical Communications,* vol. 52, pp. 3883-3886, 2016.

[5] M. I. Lucío, A. H. Montoto, E. Fernández, S. Alamri, T. Kunze, M.-J. Bañuls, *et al.*, "Label-free detection of C-Reactive protein using bioresponsive hydrogel-based surface relief diffraction gratings," *Biosensors and Bioelectronics,* vol. 193, p. 113561, 2021.

[6] Q. Fu, Z. Wu, J. Li, Z. Wu, H. Zhong, Q. Yang, *et al.*, "Quantitative assessment of disease markers using the naked eye: point-of-care testing with gas generation-based biosensor immunochromatographic strips," *Journal of nanobiotechnology,* vol. 17, pp. 1-10, 2019.

[7] Y. Panraksa, A. Apilux, S. Jampasa, S. Puthong, C. S. Henry, S. Rengpipat, *et al.*, "A facile one-step gold nanoparticles enhancement based on sequential patterned lateral flow immunoassay device for C-reactive protein detection," *Sensors and Actuators B: Chemical,* vol. 329, p. 129241, 2021.
